# Targeted therapy in lung cancer: Are we closing the gap in years of life lost?

**DOI:** 10.1002/cam4.4703

**Published:** 2022-03-22

**Authors:** David J. Benjamin, Alyson Haslam, Jenny Gill, Vinay Prasad

**Affiliations:** ^1^ Division of Hematology/Oncology, Department of Medicine University of California, Irvine Orange California USA; ^2^ Department of Epidemiology and Biostatistics University of California, San Francisco San Francisco California USA

**Keywords:** driver mutation, non‐small cell lung cancer, targeted therapy, years of life lost

## Abstract

**Purpose:**

Patients with non‐small cell lung cancer (NSCLC) that harbor driver mutations are associated with a cancer diagnosis at a younger age. While targeted therapies provide deep remissions and durable benefit in a subset of patients, it is unclear whether targeted therapies bridge the gap in years of life lost (YLL) in these younger NSCLC patients with targetable mutations in comparison to generally older NSCLC patients without actionable driver mutations.

**Materials and Methods:**

Retrospective cross‐sectional study using landmark trials leading to the approval of targeted therapies in NSCLC with actionable mutations. We evaluated all targeted therapies as well as chemotherapy and IO regimens for the treatment of NSCLC through FDA Oncology Announcements and NCCN Guidelines for NSCLC (version 4.2021).

**Results:**

We estimated the YLL for each driver mutation, cumulative median duration of response (DOR) with targeted therapies by mutation type, and percentage of estimated improvement in YLL from NSCLC targeted therapies. The median ages at diagnosis (in years) for patients whose tumors express targetable mutations were: 47.6 (*NTRK*); 52.0 (*ALK*); 62.0 (*HER2*); 57.0 (*ROS1*); 61.4 (*RET*); 63.0 (*BRAF*); 69.0 (*EGFR*); and 72.0 (*MET*). For comparison, the median age at diagnosis for patients without driver mutations, regardless of PD‐L1 status was 71 years. The median DOR (in years) for patients whose tumors express the same mutations include: 0.9 (*NTRK*); 3.9 (*ALK*); 0.6 (*HER2*); 6.2 (*ROS1*); 2.2 (*RET*); 1.5 (*BRAF*); 3.1 (*EGFR*); and 2.4 (*MET*). The median DOR for patients without driver mutations was 1.2 years. The cumulative estimated survival time (years; median age at diagnosis plus the median DOR or OS) for patients whose tumors express targetable mutations were: 48.5 (*NTRK*); 55.9 (*ALK*); 62.6 (*HER2*); 63.2 (*ROS1*); 63.6 (*RET*); 64.5 (*BRAF*); 72.1 (*EGFR*); and 74.4 (*MET*). The cumulative estimated survival time for patients without driver mutations, regardless of PD‐L1 status, was 72.2 years of age. We calculated the number of years NSCLC is diagnosed earlier in patients with targetable mutations as follows: 23.4 (*NTRK*), 19 (*ALK*), 14 (*ROS1*), 11 (*EGFR*), 9.6 (*RET*), 9 (*HER2*), and 8 (*BRAF*). The percent difference (%) ameliorated in YLL by mutation type is as follows: 44.3 (*ROS1*), 28.2 (*EGFR*), 22.9 (*RET*), 20.5 (*ALK*), 18.8 (*BRAF*), 6.4 (*HER2*), and 3.7 (*NTRK*).

**Conclusion:**

Although targeted therapies have paved the way for significant progress toward providing a survival benefit to many young patients with advanced NSCLC with actionable mutations, it is evident that these therapies still leave a wide gap in the YLL in these younger patients compared to generally older individuals with advanced NSCLC without targetable mutations.

## Graphical abstract

Patients with NSCLC that harbor driver mutations are diagnosed at a younger age than patients with NSCLC without actionable mutations, and have a significant burden of years of life lost (YLL). The median duration of response (DOR) is generally longer with targeted therapies compared to conventional chemotherapy and immunotherapy (IO). Despite durable responses, targeted therapies do not close the gap in YLL for patients with NSCLC that harbors driver mutations. More novel therapeutics are required to close the gap in YLL for patients with NSCLC with actionable mutations.

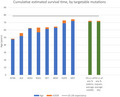

## 1 INTRODUCTION

Lung cancer is the second most common cancer among men and women in the United States, and the leading cause of cancer‐related death (1). Lung cancer is further classified into small cell lung cancer and non‐small cell lung cancer (NSCLC). For several decades, the primary systemic treatment of advanced NSCLC remained chemotherapy until the advent of tyrosine kinase inhibitor (TKI) therapy following the discovery of several driver mutations such as epidermal growth factor receptor (*EGFR*), anaplastic lymphoma kinase (*ALK*), and *ROS1*. The Cancer Genome Atlas performed molecular profiling of tissue from 230 lung adenocarcinomas and found the prevalence of targetable mutations as follows: *EGFR* (11.3%), *ALK* (1.3%), *ROS1* (1.7%), *MET* (4.3%), *RET* (0.9%), *BRAF* (7.0%), and *ERBB2/HER2* (1.7%) (2). As such, targeted therapies may benefit a growing proportion of patients with advanced NSCLC.

Studies have suggested that driver mutations are associated with a cancer diagnosis at a younger age (3). For example, patients with *ALK*‐positive tumors have a median age of 52 years at the time of diagnosis (4). Conversely, according to SEER data, the median age of patients with lung cancer at the time of diagnosis is 71 years old (5). It is undeniable that targeted therapies provide deep remissions and durable benefit in a subset of patients, but it remains unclear whether targeted therapies bridge the gap in years of life lost (YLL) in these younger NSCLC patients with targetable mutations in comparison to generally older NSCLC patients without actionable driver mutations.

For example, a 71‐year‐old patient with a significant history of tobacco use may be diagnosed with advanced NSCLC adenocarcinoma without any driver mutation, and be treated with standard‐of‐care chemotherapy and immunotherapy (IO; carboplatin, pemetrexed and pembrolizumab) based on KEYNOTE‐189 (6). The patient can expect a median of 1.26 years duration of response (DOR) based on current clinical trial data. Given US life expectancy, the patient would experience 6.4 years of life lost (78.7–72.3 = 6.4).

In contrast, the average age of patients diagnosed with *ALK*‐positive NSCLC is 52 years. Under optimistic assumptions, such a patient may experience a duration of response for 3.9 years from sequential TKI use. In such a scenario, the patient would lose 22.8 years of life (78.7–55.9 = 22.8). In this study, we sought to estimate the years of life lost with current NSCLC treatment based on molecular or biomarker subgroup.

## 2 METHODS

### 2.1 Overview

We sought to estimate the potential treatment responses associated with targeted therapies for actionable driver mutations in NSCLC, as well as treatment responses associated with standard‐of‐care regimens involving chemotherapy and IO. Per policies at the University of California, the study was not submitted for institutional review board approval at either institution because all data are publicly available and because it did not involve health records. The study was conducted between January 2021 and March 2021.

### 2.2 Dataset

We evaluated all targeted therapies as well as chemotherapy and IO regimens for the treatment of NSCLC that have been approved by the U.S. Food and Drug Administration (FDA) through March 3, 2021. To this end, we searched for landmark studies in NSCLC as well as for drug approvals through FDA oncology announcements. We also searched standard‐of‐care treatment options for actionable mutations in NSCLC and treatment options based on PDL‐1 status for patients with no targetable mutations by examining the National Comprehensive Cancer Network (NCCN) Guidelines for NSCLC (Version 4.2021, March 3, 2021). We then conducted searches on PubMed to further evaluate clinical trial results for each actionable mutation and targeted therapy (e.g., “*ALK*”+“alectinib”+“NSCLC”+“duration of response”). Data extracted from each clinical trial includes median DOR, and if not available, median OS, as well as the median age of enrolled patients. In addition, in order to calculate YLL, we conducted searches on PubMed (e.g. “median age”+“diagnosis”+“EGFR”) to evaluate the median age of diagnosis in NSCLC patients with or without actionable mutations based on clinical trial data and community‐based data. For comparison, we identified KEYNOTE‐189 and KEYNOTE‐407 as the landmark trials regarding standard‐of‐care for advanced NSCLC without a driver mutation of adenocarcinoma histology and squamous cell histology, respectively.

In addition to targeted therapy, the introduction of immunotherapy (IO) has revolutionized the treatment landscape of advanced NSCLC. In 1973, the relative 5‐year survival rate of patients with lung cancer was 10.7% (7). In 2019, 5‐year results from the phase IB KEYNOTE‐001 trial estimated a 23.2% 5‐year overall survival (OS) in treatment‐naïve patients with NSCLC who received pembrolizumab monotherapy (8). In 2020, updated analysis from the ALEX trial demonstrated a 5‐year OS rate of 62.5% in patients taking alectinib for *ALK*‐mutated NSCLC (9). Multiple such studies over the past decade evaluating targeted therapy and IO in NSCLC have demonstrated high response rates and durable responses compared to conventional chemotherapy alone, leading to optimism in treatments based on molecular subtypes and checkpoint inhibition.

### 2.3 Statistical analysis

We defined treatment response as median DOR as reported in clinical trials. We used 78.7 years as the median life expectancy in the United States (10). Although life expectancy changes over time, and is contingent on a person's achieved age, we used the overall U.S. life expectancy for all calculations for simplicity.

To estimate the potential treatment effects of targeted therapies as well as standard‐of‐care chemotherapy and IO, we summed the median DOR or median OS for each targeted therapy that is approved or used for a corresponding targetable action or chemotherapy and IO regimen for NSCLC without an actional mutation. We summed the responses with each therapy given that patients generally receive sequential treatment with targeted therapies.

To estimate the YLL for each driver mutation, we calculated the difference in the median age of cancer diagnosis for patients with the respective driver mutation and the average age of cancer diagnosis for patients who do not have a driver mutation. For the latter value, we used the median age of diagnosis of NSCLC provided by SEER data (5).

Finally, for each driver mutation, we calculated a percentage of estimated improvement from NSCLC targeted therapies; in other words, we calculated the percent (%) difference of YLL ameliorated by targeted therapies. We divided the number of years gained with targeted therapies according to each mutation type by the number of years NSCLC was diagnosed by mutation type. For example, the cumulative mDOR with EGFR inhibitors is 3.775 years. Patients with *EGFR*‐mutated NSCLC are diagnosed 11 years earlier compared to those without an actionable mutation. Therefore, the percent difference in YLL that is ameliorated by *EGFR*‐targeted therapies is 34.3% (3.775/11*100). Descriptive analyses were conducted using Excel (Microsoft Corporation).

## 3 RESULTS

**FIGURE 1:**
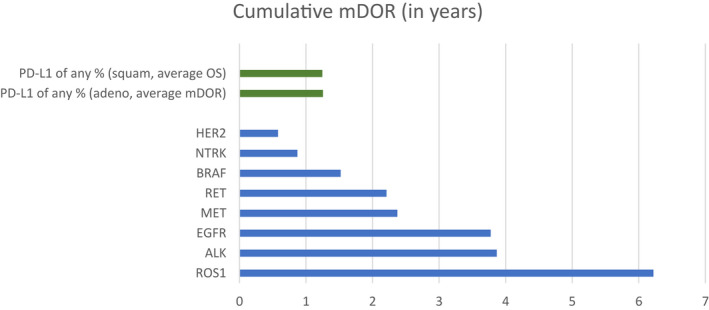
Cumulative median duration of response (DOR, in years) with all targeted therapies by mutation

Nineteen targeted therapies for the treatment of NSCLC with actionable mutations were approved by the FDA or recommended per NCCN guidelines through March 1, 2021. These therapies include erlotinib, afatinib, gefitinib, osimertinib and dacomitinib for *EGFR* mutation positive NSCLC; crizotinib, ceritinib, alectinib, brigatinib, and lorlatinib for *ALK* rearrangement positive NSCLC; crizotinib, entrectinib, and lorlatinib for *ROS1* rearrangement positive NSCLC, dabrafenib and trametinib for *BRAF V600E* mutation positive NSCLC; larotrectinib and entrectinib for *NTRK* gene fusion positive NSCLC; capmatinib, crizotinib and tepotinib for *MET* exon 14 skipping NSCLC; and selpercatinib and pralsetinib for *RET* rearrangement positive NSCLC. The antibody conjugate trastuzumab deruxtecan has received FDA approval for the treatment of *HER2*‐mutated NSCLC. Additional therapies that have been studied in *HER2*‐mutated NSCLC include the TKI pyrotinib as well as HER2 antibody conjugate pertuzumab Figure 1.

### 3.1 Median age

**FIGURE 2:**
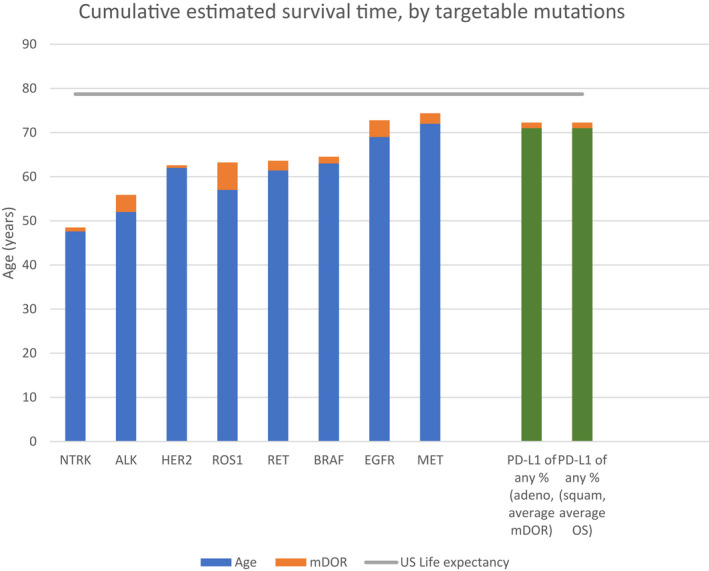
Estimated Survival (median age of diagnosis+cumulative median duration of response) by targetable mutation

The median ages at diagnosis (in years) for patients whose tumors express targetable mutations were (Figure 2): 47.6 (*NTRK*); 52.0 (*ALK*); 62.0 (*HER2*); 57.0 (*ROS1*); 61.4 (*RET*); 63.0 (*BRAF*); 69.0 (*EGFR*); and 72.0 (*MET*) (4, 11, 12, 13, 14, 15, 16, 17). For comparison, the median age at diagnosis for patients without driver mutations, regardless of PD‐L1 status was 71 years of age. The median DOR (in years) for patients whose tumors express the same mutations include: 0.9 (*NTRK*); 3.9 (*ALK*); 0.6 (*HER2*); 6.2 (*ROS1*); 2.2 (*RET*); 1.5 (*BRAF*); 3.775 (*EGFR*); and 2.4 (*MET*). The median duration of response for patients without driver mutations, regardless of PD‐L1 status was 1.2 years. The cumulative estimated survival time (years; median age at diagnosis plus the median DOR or OS) for patients whose tumors express targetable mutations were: 48.5 (*NTRK*); 55.9 (*ALK*); 62.6 (*HER2*); 63.2 (*ROS1*); 63.6 (*RET*); 64.5 (*BRAF*); 72.1 (*EGFR*); and 74.4 (*MET*). The cumulative estimated survival time for patients without driver mutations, regardless of PD‐L1 status was 72.2 years of age.

### 3.2 Earlier onset of NSCLC

With the exception of *MET* exon 14 skipping mutation NSCLC, patients who have actionable mutations tend to be diagnosed at an earlier age compared to patients with no targetable mutation. We defined 71 years as the median age of diagnosis for NSCLC without a targetable mutation based on SEER data in order to determine how much earlier patients with targetable mutations are diagnosed. We calculated the number of years NSCLC is diagnosed earlier in patients with targetable mutations as follows: 23.4 (*NTRK*), 19 (*ALK*), 14 (*ROS1*), 11 (*EGFR*), 9.6 (*RET*), 9 (*HER2*), and 8 (*BRAF*). Patients with MET exon 14 skipping mutations have a median age of diagnosis of 72 years, and therefore, are generally diagnosed 1 year later than a patient without a targetable mutation. It is evident that patients with several of these targetable mutations such as *NTRK*, *ALK*, *ROS1*, and *EGFR* have a considerable burden of YLL due to diagnosis at a much earlier age than a patient without the targetable mutation. The median age of diagnosis of NSCLC and the number of years earlier that NSCLC is diagnosed in patients with actionable mutations is presented in Table S2.

### 3.3 Median duration of response or overall survival

The median DOR (in months) for approved therapies that target *EGFR* mutations include: osimertinib = 17.2 (18), afatinib = 11.1 (19), gefitinib = 8.5 (18), and erlotinib = 8.5 (18). Updated analysis from the ARCHER 1050 trial did not report a median DOR associated with dacomitinib therapy; however, the median OS associated with treatment was 34.1 months (20). As it is not possible to add mDOR to mOS, dacomitinib was not used to estimate the overall response from approved EGFR inhibitors if used sequentially. The median DOR for approved therapies for *ALK* rearrangement positive NSCLC include: crizotinib = 11.3 (21), alectinib = 11.2 (22), and ceritinib = 23.9 (23). Analysis from more recent studies such as the ALEX trial, ASCEND‐3 trial, ALTA‐1 L trial and CROWN trial evaluating alectinib, certinib, brigatinib, and lorlatinib, respectively, do not report mDOR (24, 25, 26, 27). The median DOR for approved therapies for *ROS1* rearrangement positive NSCLC include: crizotinib = 24.7 (28), entrectinib = 24.6 (29), and lorlatinib = 25.3 (30). Dabrafenib/trametinib combination therapy is approved for the treatment of *BRAF V600E* mutation‐positive NSCLC, and is associated with a median OS of 18.2 months (31). Entrectinib and larotrectinib are both approved in the treatment of *NTRK* gene fusion NSCLC. Although the median DOR/OS is unavailable for larotrectinib, the median DOR associated with entrectinib is 11.1 months (32, 33). The median DOR for approved therapies that treat *MET* exon 14 skipping mutation NSCLC include: capmatinib = 8.3, crizotinib = 9.1, and tepotinib = 11.1 (34, 35, 36). The median DOR for therapies approved in treating *RET* rearrangement positive NSCLC include: selpercatinib = 17.5 and pralsetinib = 9.0 (37, 38). Trastuzumab deruxtecan is approved for the treatment of *HER2*‐mutated NSCLC; however, median DOR data has not yet been reached (39). Although not FDA approved, the TKI pyrotinib has been studied in this patient population and is associated with a median DOR of 6.9 months (40).

For patients with NSCLC adenocarcinoma subtype without a targetable mutation, the median DOR with chemotherapy and IO by PDL‐1 status is as follows: 15.1 (PDL‐1 > 50%), 12.9 (PDL‐1 1%–49%), and 10.8 (PDL‐1 < 1%) (41). For patients with NSCLC squamous cell subtype without a targetable mutation, the median OS with chemotherapy and IO by PDL‐1 status is as follows: 14.0 (PDL‐1 1%–49%) and 15.9 (PDL‐1 < 1%) (42). The median OS in patients with squamous cell subtype and PDL‐1 > 50% has not yet been reached. Median DOR and OS data are presented in Table S1, in addition to trial information.

### 3.4 Amelioration of YLL

**FIGURE 3:**
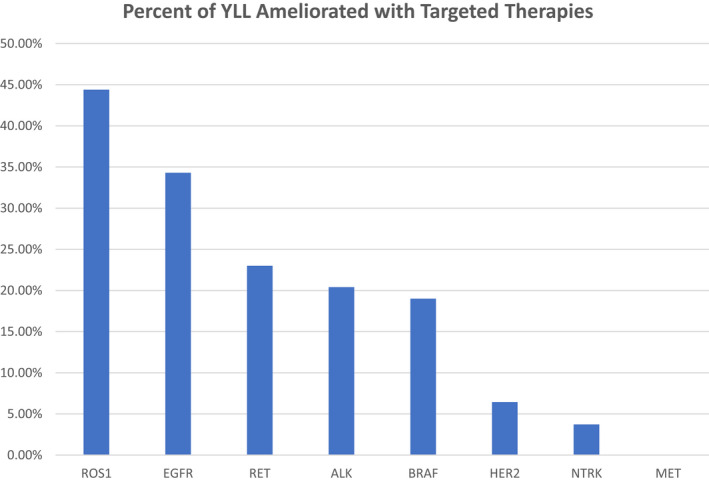
Percent of years of life lost (YLL) ameliorated with targeted therapies

The percent difference (%) ameliorated in YLL by mutation type is as follows: 44.3 (*ROS1*), 34.3 (*EGFR*), 22.9 (*RET*), 20.5 (*ALK*), 18.8 (*BRAF*), 6.4 (*HER2*), and 3.7 (*NTRK*). The percent difference of YLL ameliorated by targeted therapies is presented in Figure 3 and Table S3.

## 4 DISCUSSION

To our knowledge, this is the first study to evaluate the estimated survival as well as estimated YLL in patients treated with targeted therapies for advanced NSCLC that harbor actionable oncogenic mutations including *EGFR*, *ALK*, *ROS1*, *MET*, *RET*, *HER2*, *BRAF*, and *NTRK*. The advent of therapies targeting actionable mutations has generated considerable enthusiasm in the treatment for metastatic NSCLC, which has long carried a poor prognosis. While there is justified reason for the enthusiasm surrounding several of these therapies that provide durable responses not seen with conventional chemotherapy, the progress in targeted therapeutic options must be evaluated in the context of the demographics of these patients with actionable mutations. It is evident that patients with actionable mutations, with the exception of *MET* exon 14 skipping who are diagnosed at a median age of 72 years of age, tend to be younger than patients without actionable mutations. As such, most of these generally younger patients with targetable mutations, except for those with *MET* exon 14 skipping mutations, have several YLL as they do not reach the general population's median life expectancy even if they were to receive all known approved targeted therapies for the mutation in their NSCLC. In other words, even under the most optimistic scenario with all targeted therapies employed, many of these younger patients still carry a greater burden of YLL compared to those older patients with NSCLC without targetable mutations. Under the most ideal circumstances, a patient with a *ROS1* tumor will have less than half (44.3%) of the YLL ameliorated from *ROS1* targeted therapies, followed by patients with *EGFR* mutations that are treated with EGFR inhibitors (34.3%), while patients whose tumors harboring *HER2* (6.4%) and *NTRK* (3.7%) mutations will have an amelioration of approximately or less than 5% of the YLL despite taking all approved targeted therapies for their corresponding mutation. It is therefore evident that despite advancements in targeted therapeutics, patients with actionable mutations still suffer a considerable amount of YLL.

There are several limitations associated with this observational study. First, our analysis may overestimate the therapeutic benefit of each targeted therapy. We used clinical trial data for median DOR and median OS to estimate the potential added survival benefit for the average patient with a targetable NSCLC mutation. However, this does not account for real‐world conditions that generally do not mirror the settings of a large clinical trial and where median DOR and median OS are not replicated. Second, not all clinical trials estimated median DOR or median OS for several therapies including dacomitinib for *EGFR*‐mutated NSCLC, alectinib for *ALK*‐mutated NSCLC, and larotrectinib for *NTRK*‐mutated NSCLC. As such, it was unclear how to provide a reasonable estimate for median DOR or median OS for these therapies. Third, we could not account for changes in treatment due to TKI resistance mechanisms and where patients could not be treated with a different generation TKI. Fourth, while we included some therapies that had off‐label use for metastatic NSCLC, such as pyrotinib for *HER2*‐mutated NSCLC, we were unable to include all potential therapies that are used off‐label for NSCLC with actionable mutations. There is no database that encompasses all therapies that are used in treatment of patients, as clinical practices vary between academic centers as well as between community‐based practices.

Although targeted therapies have paved the way for significant progress toward providing a survival benefit to many young patients with advanced NSCLC with actionable mutations, it is evident that these therapies still leave a wide gap in the YLL in these younger patients compared to generally older individuals with advanced NSCLC without targetable mutations. This study, therefore, provides a new framework wherein medical oncologists, other oncology care providers and patients can contextualize the enthusiasm for approved targeted therapies in juxtaposition of the generally earlier diagnosis among patients whose NSCLC harbor actionable mutations. The misconception that patients are “fortunate” or “lucky” to have targetable mutations can be misleading as although these targeted therapies may prolong life compared to conventional chemotherapy, there still remains a significant YLL in these younger individuals. The cumulative YLL of these individuals across the globe has both short‐ and long‐term multi‐level consequences on patient's families, communities, and society on a multitude of levels. The progress made in targeted therapies for advanced NSCLC is commendable and deserves praise, but it is unmistakable that considerable progress is still to be made before patients can bridge the gap in YLL.

## CONFLICT OF INTEREST

Vinay Prasad Disclosures. (Research funding) Arnold Ventures (Royalties) Johns Hopkins Press, Medscape, MedPage (Consulting) UnitedHealtcare. (Speaking fees) Evicore. New Century Health (Other) Plenary Session podcast has Patreon backers.

## AUTHOR CONTRIBUTION

**David J. Benjamin** carried out conceptualization, methodology, investigation, formal analysis, validation, writing—original draft, and writing—review and editing of the manuscript. **Alyson Haslam** carried out investigation, validation, formal analysis, writing—review and editing of the manuscript. **Jenny Gill** was involved in investigation, validation, visualization, formal analysis, and writing—review and editing of the manuscript. **Vinay Prasad** carried out conceptualization, methodology, formal analysis, writing—original draft, writing—review and editing, supervision, and funding acquisition of the manuscript.

## ETHICAL APPROVAL STATEMENT

Per policies at the University of California, the study was not submitted for institutional review board approval at either institution because all data are publicly available and because it did not involve health records.

## Supporting information


Tables S1‐S3

## Data Availability

The data that support the findings of this study are available in landmark studies in NSCLC as well as for drug approvals through FDA oncology announcements. These data were derived from the following resources available in the public domain: landmark trials all available through PubMed.
