# Supplementary material for: Targeted therapy in lung cancer: Are we closing the gap in years of life lost?
Source: Cancer Med. 2022 Mar 22;11(18):3417–24. doi: 10.1002/cam4.4703 (PMC9487872; doi:10.1002/cam4.4703)
Supplement: Supplementary file 1 — Tables S1‐S3 [file CAM4-11-3417-s001.docx]

**Supplementary Appendix**

**Table 1. Targeted therapies and mDOR associated with each therapy based off mutation**

| **Actionable Mutation; if no target, PDL-1 status as indicated** | **Drug(s)** | **Median DOR (months); if not available, median OS as indicated** |
| --- | --- | --- |
| **EGFR** | Osimertinib | 17.2 |
|  | Erlotinib | 8.5 |
|  | Gefitinib | 8.5 |
|  | Afatinib | 11.1 |
|  | Dacomitinib | 34.1 (median OS) |
| **ALK** | Crizotinib | 11.3 months |
|  | Ceritinib | 23.9 months |
|  | Alectinib | 11.2 months |
|  | Brigatinib | Not reached (median DOR) per ALTA-A1; however, retrospective BRIGALK shows median OS of 17.2 |
|  | Lorlatinib | Data unavailable |
| **ROS1** | Crizotinib | 24.7 |
|  | Entrectinib | 24.6 |
|  | Lorlatinib | 25.3 |
| **BRAF V600E** | Dabrafenib and trametinib | 18.2 (median OS for combination therapy) |
| **NTRK** | Larotrectinib | Not reached (median DOR and median OS) |
|  | Entrectinib | 10.4 |
| **MET** | Capmatinib | 8.31 |
|  | Crizotinib | 9.1 |
|  | Tepotinib | 11.1 |
| **RET** | Selpercatinib | 17.5 |
|  | Pralsetinib | 9.0 |
| **HER2** | Trastuzumab deruxtecan | Not reached (median DOR) |
|  | Pyrotinib | 6.9 |
|  | Pertuzumab | Data unavailable |
| **PDL-1 status 50% (adenocarcinoma)** | Carboplatin + Pemetrexed + Pembrolizumab | 15.1 |
| **PDL-1 status <1% (adenocarcinoma)** | Carboplatin + Pemetrexed + Pembrolizumab | 10.8 |
| **PDL-1 status 50% (squamous cell)** | Carboplatin + Paclitaxel + Pembrolizumab | Not reached (median OS) |
| **PDL-1 status <1% (squamous cell)** | Carboplatin + Paclitaxel + Pembrolizumab | 15.9 (median OS) |

**Table 2: how many years earlier does NSCLC appear in patients with driver mutation than NSCLC without a driver mutation (defined as 71 years per SEER data)**

| **NSCLC driver mutation** | **Median onset (age) at diagnosis** | **Number of years NSCLC is diagnosed earlier** |
| --- | --- | --- |
| **EGFR** | 60 years | 11 years |
| **ALK** | 52 years | 19 years |
| **ROS1** | 57 years | 14 years |
| **BRAF** | 63 years | 8 years |
| **NTRK** | 47.6 years | 23.4 years |
| **MET** | 72 years | 1 year later |
| **RET** | 61.4 years | 9.6 years |
| **HER2** | 62 years | 9 years |

**Table 3: percent difference in number of years NSCLC is diagnosed earlier ameliorated with targeted drugs**

| **NSCLC driver mutation** | **Number of years NSCLC is diagnosed earlier** | **Number of years gained with all targeted therapies** | **Percent difference ameliorated with targeted drugs** |
| --- | --- | --- | --- |
| **EGFR** | 11 years | 3.775 | 34.3% |
| **ALK** | 19 years | 3.866 | 20.4% |
| **ROS1** | 14 years | 6.22 | 44.4% |
| **BRAF** | 8 years | 1.52 | 19% |
| **NTRK** | 23.4 years | 0.87 | 3.72% |
| **MET** | 1 year later | 2.373 | N/A |
| **RET** | 9.6 years | 2.21 | 23.0% |
| **HER2** | 9 years | 0.58 | 6.44% |
